# Single Image Haze Removal from Image Enhancement Perspective for Real-Time Vision-Based Systems

**DOI:** 10.3390/s20185170

**Published:** 2020-09-10

**Authors:** Dat Ngo, Seungmin Lee, Quoc-Hieu Nguyen, Tri Minh Ngo, Gi-Dong Lee, Bongsoon Kang

**Affiliations:** 1 Department of Electronics Engineering, Dong-A University, Busan 49315, Korea; datngo@donga.ac.kr (D.N.); 1672885@donga.ac.kr (S.L.); 1972195@donga.ac.kr (Q.-H.N.); gdlee@dau.ac.kr (G.-D.L.); 2 Faculty of Electronics and Telecommunication Engineering, The University of Danang—University of Science and Technology, Danang 550000, Vietnam; nmtri@dut.udn.vn

**Keywords:** haze removal, real-time processing, detail enhancement, multiple-exposure image fusion, adaptive tone remapping, field programmable gate array

## Abstract

Vision-based systems operating outdoors are significantly affected by weather conditions, notably those related to atmospheric turbidity. Accordingly, haze removal algorithms, actively being researched over the last decade, have come into use as a pre-processing step. Although numerous approaches have existed previously, an efficient method coupled with fast implementation is still in great demand. This paper proposes a single image haze removal algorithm with a corresponding hardware implementation for facilitating real-time processing. Contrary to methods that invert the physical model describing the formation of hazy images, the proposed approach mainly exploits computationally efficient image processing techniques such as detail enhancement, multiple-exposure image fusion, and adaptive tone remapping. Therefore, it possesses low computational complexity while achieving good performance compared to other state-of-the-art methods. Moreover, the low computational cost also brings about a compact hardware implementation capable of handling high-quality videos at an acceptable rate, that is, greater than 25 frames per second, as verified with a Field Programmable Gate Array chip. The software source code and datasets are available online for public use.

## 1. Introduction

Images or videos taken outdoors usually suffer from an apparent loss of contrast and details owing to the inevitable adverse effects of bad weather conditions. Spatially varying degradation sharply decreases the performance of computer vision and consumer applications, such as surveillance cameras, autonomous driving vehicles, traffic sign detecting systems, and notably face recognition, which is widely adopted on smartphones and Internet-of-Things devices [1]. Haze removal, also known as dehazing or defogging, addresses this problem by eliminating the undesirable effects of the transmission medium and restoring clear visibility.

In general, haze removal algorithms fall into two categories—single- and multiple-image algorithms. Even though the latter is no longer of interest to researchers, its superior performance is worthy of attention. According to Rayleigh’s scattering law [2], the scattering of incoming light in the atmosphere is inversely proportional to the wavelength, and therein lies the cause of the wavelength-dependent haze distribution. Thus, the polarizing filter, which allows light waves of a specific polarization to pass through while reflecting light waves of other polarizations, was widely used in photography as a first attempt to remove haze. However, this polarization filtering technique alone could not remove the hazy effects because the atmospheric scattering phenomenon appeared at different wavelengths. As a result, Schechner et al. [3] developed a method that took at least two images captured under different polarization to estimate the airlight and the scaled depth. Subsequently, they reversed the hazy image formation process to restore the original haze-free scene radiance. On the other hand, Narasimhan and Nayar [4] proposed an approach that could function properly with as few as two images taken in different weather conditions, and Kopf et al. [5] exploited the existing georeferenced digital terrain and urban models to remove haze from outdoor images. Notwithstanding the impressive dehazing performance, researchers were apathetic about multiple-image algorithms due to the difficulties in the input acquisition process, consequently leading to the rapid development of single-image haze removal algorithms.

Researchers usually approach removing haze from a single input image from two perspectives: image restoration and image enhancement. In the former, a physical model, referred to as Koschmieder atmospheric scattering model [6], describes the formation of hazy images in the atmosphere. The incoming light is not only scattered by microscopic particles like dust or water droplets, but it is also attenuated when traversing the transmission medium. Therefore, solving the Koschmieder model with the two unknown variables representing the phenomena mentioned earlier is considered problematic. He et al. [7] proposed a dark channel prior (DCP), which states that there exist some pixels whose intensity is very low in at least one color channel of non-sky local patches. DCP provided an efficient way to estimate the patch-based extinction coefficients of the transmission medium. Subsequently, the computationally expensive soft-matting [8] was employed to suppress the block artifacts, giving rise to the DCP’s time-consuming drawback. They after that proposed a multi-function guided image filter [9], which could replace the soft-matting for accelerating the DCP at the cost of a specific degradation in image quality. The DCP has also been developed in many directions to overcome its shortcomings of slow processing rate and unpredictable performance in sky regions [10,11,12,13,14,15,16]. A fast dehazing method proposed by Kim et al. [15] and its corresponding 4K-capable intellectual property (IP) presented in Reference [17] are cases considered. By exploiting the modified hybrid median filter, which possesses excellent edge-preserving characteristics, to estimate the atmospheric veil, their method eliminated the need for soft-matting refinement, resulting in considerably faster processing speed, albeit a slight degradation in image quality. Based on the observation that the scene depth positively correlates with the difference between the image’s saturation and brightness, Zhu et al. [18] developed a linear model known as color attenuation prior (CAP). They then estimated its parameters in a supervised learning manner. Since the depth is exponentially proportional to the extinction coefficients of the transmission medium, CAP provided a fast way to solve the Koschmieder model. However, according to a detailed investigation conducted by Ngo et al. [19], it suffered from a few shortcomings, such as background noise, color distortion, and reduced dynamic range. Tang et al. [20] utilized another machine learning technique called random forest regression to calculate the extinction coefficients from a set of multi-scale image features including DCP, local maximum contrast, hue disparity, and local maximum saturation. Ngo et al. [21] exploited the simplex-based Nelder-Mead optimization to find the optimal transmission map. They additionally devised an adaptive atmospheric light to account for the heterogeneity of lightness. Although the dehazing power of the methods proposed by Tang et al. [20] and Ngo et al. [21] is quite impressive, several practical difficulties resulted owing to their highly expensive computations. Recently, deep learning techniques, notably convolutional neural networks (CNNs), have been used to learn the unknown variables of the Koschmieder model from the collected data. Cai et al. [22] developed a shallow-but-efficient CNN dubbed DehazeNet, taking a hazy image and producing its corresponding extinction coefficients. Other studies [23,24,25,26] have attempted to improve performance by either increasing the receptive field via deeper networks or developing a more sophisticated loss function as a surrogate for the universally used mean squared error. However, they all share the same problem of lacking a real training dataset comprising pairs of hazy and haze-free images. This drawback imposes a limit on their dehazing power. Interested readers are referred to a comprehensive review provided by Li et al. [27].

Since estimating two unknown variables in the Koschmieder model is somewhat computationally expensive, researchers have attempted to dehaze images employing image enhancement techniques. Instead of relying on a physical model, they first attempted to enhance low-level features such as contrast, sharpness, and brightness for alleviating the adverse effects of haze. Low-light stretch [28], unsharp masking [29,30,31], and histogram equalization [32,33] are cases considered. Nonetheless, since these methods did not take the underlying cause of haze-relevant distortion into account, they were merely appropriate to images obscured by thin haze. With current efforts in exploiting image fusion techniques, this category of haze removal algorithms has become haze-aware. Ancuti et al. [34] developed a method where the fusion followed a multi-scale manner with corresponding weight maps derived from luminance, chrominance, and salience information. Choi et al. [35] investigated haze-relevant features such as contrast energy, image entropy, normalized dispersion, and colorfulness, to name but a few. They then developed a more sophisticated weighting scheme for selectively blending regions with good visibility to the restored image. Galdran [36] also removed the hazy effects utilizing multi-scale fusion, but weight maps solely comprised contrast and saturation. As haze-relevant image features constituted the weight maps, these approaches achieved improved performance and extended their applicability to a wide variety of images with different haze density. However, the multi-scale fusion process, represented by the Laplacian pyramid, is quite expensive because of image buffers and line memories that are resulted from up- and down-sampling operations.

**Figure 1:**
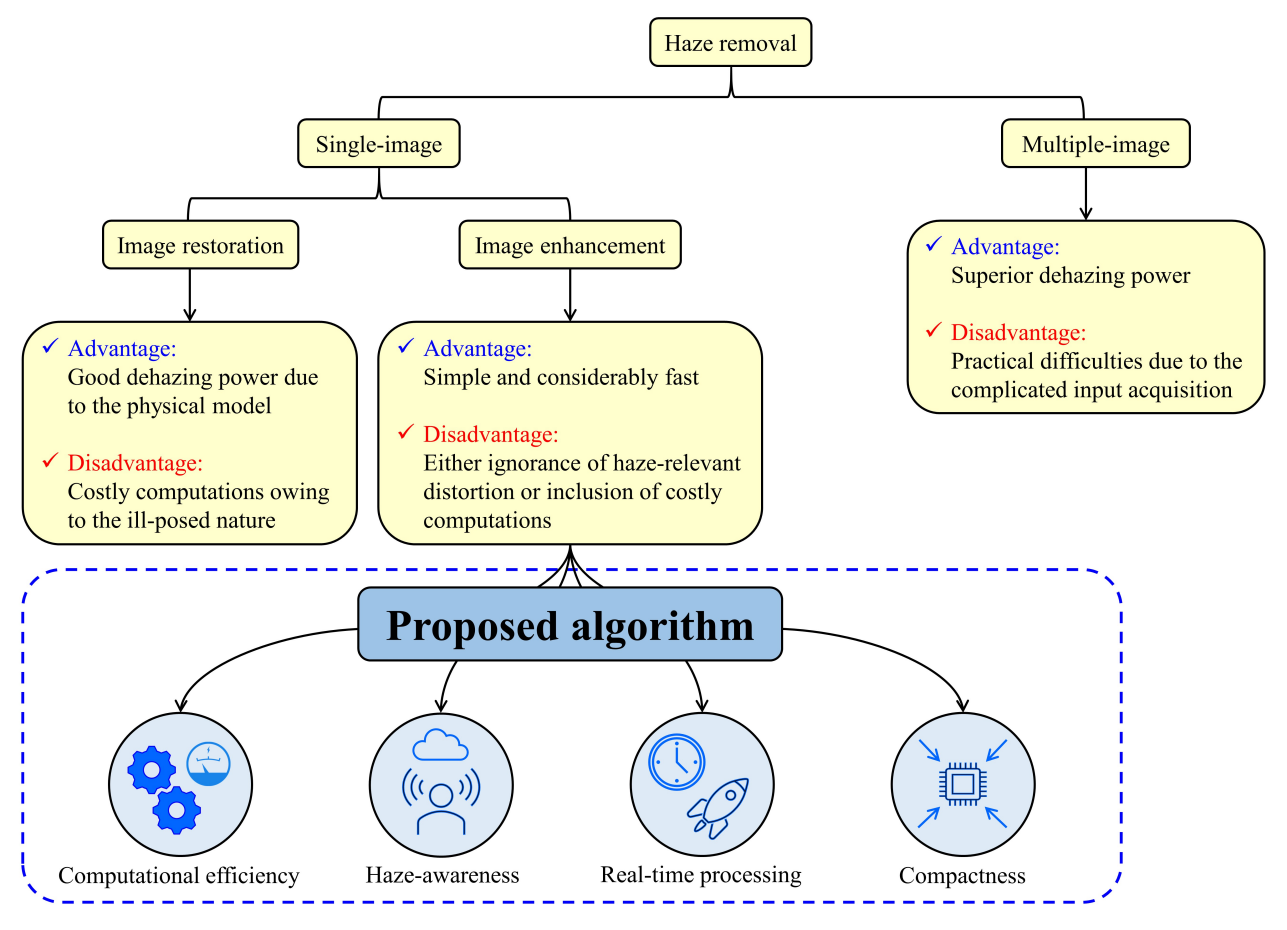
A general classification of haze removal algorithms.

In this paper, we present a novel and simple image enhancement-based haze removal method capable of producing satisfactory results. Based on the observation that haze often obscures image details and increases brightness, a set of detail-enhanced and under-exposed images derived from a single hazy image is employed as inputs to image fusion. The corresponding weight maps are calculated according to DCP, which is well recognized as a good haze indicator. Then, the fusion process is simply equivalent to a weighted sum of images and weight maps. Finally, a post-processing method known as adaptive tone remapping is employed for expanding the dynamic range. Thus, the proposed algorithm is computationally efficient and haze-aware, while its compact hardware counterpart is capable of handling videos in real-time. Figure 1 depicts a general classification of haze removal algorithms as a summary of this section.

The remainder of this paper is organized as follows. Section 2 introduces the Koschmieder model and explains the relation between under-exposure and haze removal. Section 3 presents the proposed algorithm by sequentially describing individual main computations. Section 4 conducts a comparative evaluation with other state-of-the-art methods. Section 5 describes a hardware architecture for facilitating real-time processing, while Section 6 concludes the paper.

## 2. Preliminaries

The Koschmieder model is used to highlight the importance of under-exposure in haze removal. This physical model describes the formation of hazy images by taking into account the atmospheric scattering phenomenon. Hence it can be exploited to derive the relation between hazy and clear images. By showing that the haze-free image contains lower intensity values than the hazy image, the ultimate goal of achieving the dehazing effect via fusing a set of under-exposed images becomes attainable.

### 2.1. Koschmieder Model

**Figure 2:**
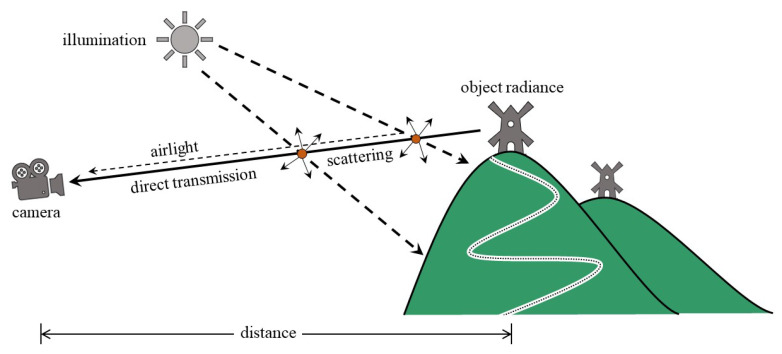
An illustration of the atmospheric scattering phenomenon.

The light waves reflected from the object, also called the object radiance, usually suffer from two main distortion types. The first one is direct attenuation and represents the gradual extinction of the object radiance in the transmission medium. In contrast, the second one is airlight and represents the scattering phenomenon when the reflected light waves encounter the atmospheric aerosols. As shown in Figure 2, they are both dependent on the distance from the observer (e.g., camera) to the object. Hence, the formation of hazy images is expressed in terms of proportionally diminishing attenuation and increasing airlight as follows:

(1) $$I\left(x\right)=J\left(x\right)\cdot e^{-\beta (\lambda )d(x)}+A\cdot \left[1-e^{-\beta (\lambda )d(x)}\right]$$

where I, J, *d*, and A denote the captured image, the clear image, the scene depth, and the global atmospheric light, respectively; *x* represents the spatial coordinates of image pixels; β stands for the extinction coefficient of the atmosphere, and λ is the wavelength. As mentioned earlier in Section 1 about multiple-image haze removal, the wavelength-dependent β(λ) causes the corresponding wavelength-dependent haze distribution. However, this dependency is widely assumed to be negligible in virtually all haze removal algorithms. Thus, by letting $t\left(x\right)\approx e^{-\beta (\lambda )d(x)}$ be the transmission map, Equation (1) is re-written as follows:

(2) $$I\left(x\right)=J\left(x\right)\cdot t\left(x\right)+A\cdot \left[1-t(x)\right]$$

### 2.2. Pertinence of Under-Exposure to Haze Removal

**Figure 3:**
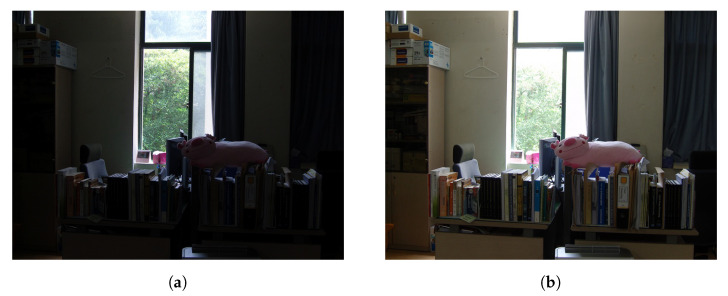
Image of different exposures: (**a**) under-exposure and (**b**) over-exposure.

The term exposure is used in photography to indicate the amount of light that reaches the electronic image sensors, and it is determined by shutter speed (i.e., exposure time) and lens aperture. Since the range of image intensities recorded is limited, for example, 256 possible intensity values of 8-bit RGB data, correct exposure is of great importance. If the exposure time is too short, an image suffers from a loss of shadow details, as shown in the desk depicted in Figure 3a. In contrast, if the digital sensors are exposed to light for too long, important bright parts of an image appear as clipped whites, as depicted in the window of Figure 3b. Thus, image fusion, also called image blending, that makes use of both under-exposed and over-exposed images is widely employed to provide a precisely exposed image. Since the atmospheric scattering phenomenon increases the amount of light entering the lens aperture, reducing the exposure time effectively restores bright details faded by haze at the cost of losing some shadow details. Hence, selectively blending clear areas from images of different exposures is similar to removing the hazy effects from the captured image.

In order to show that the deduction mentioned above is mathematically valid, we first derive another formula for the transmission map by rearranging Equation (2). Then, the exponential relation between t(x) and d(x) is used for demonstrating that d(x)∈[0,∞) leads to t(x)∈(0,1]. Applying the condition t(x)≤1 to Equation (3) results in J(x)≤I(x) because the global atmospheric light is generally larger than most image pixels. The relation between J(x) and I(x) implies that the scene radiance increases in intensity due to the atmospheric scattering phenomenon. Therefore, by blending several under-exposed images derived from the single hazy input, clear visibility can be restored.

(3) $$t\left(x\right)=\frac{I(x)-A}{J(x)-A}$$

## 3. Proposed Algorithm

**Figure 4:**
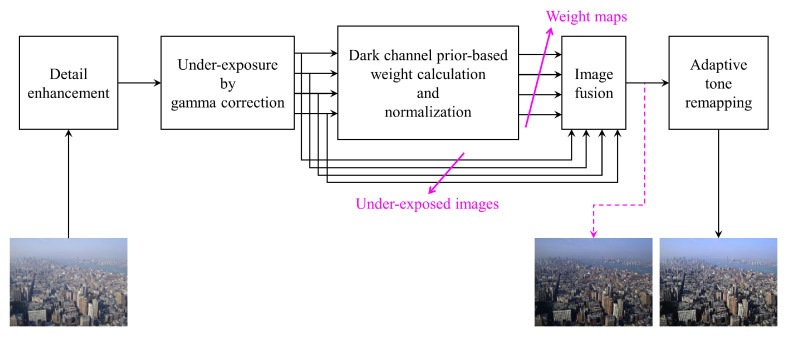
Overall block diagram of the proposed algorithm.

As under-exposing an image requires human intervention for adjusting either shutter speed or lens aperture, it cannot be attained in an automated manner. Accordingly, a simple-and-efficient technique called gamma correction is exploited to mimic the physical under-exposure. However, since gamma correction simply applies the same amount of decrease to the entire image, bright details faded by haze still remain obscure in the artificially under-exposed images. To overcome this issue, the sole input of a hazy scene is pre-processed by a detail enhancement algorithm to restored faded details. For accurately blending haze-free areas into the fused image, weight maps are first calculated with regard to dark channels and then are normalized to avoid the out-of-range problem. Nevertheless, the fused image is darker than the hazy input as a result of fusing a set of under-exposed images. Hence, a post-processing algorithm called adaptive tone remapping is employed to enhance the luminance and emphasize the chrominance. Figure 4 depicts the overall block diagram of the proposed algorithm. Individual computing processes are described in the following subsections.

### 3.1. Detail Enhancement

Detail enhancement, also called sharpness enhancement, is an image processing technique that involves three main steps: (i) decomposing the input image into background and detail signals, (ii) multiplying the latter (i.e., detail signal) by an adequate factor, and (iii) adding the enhanced details back to the background signal. However, since decomposition usually involves applying an edge-preserving filter iteratively [29,31], the hardware realization is quite cumbersome. A variant of detail enhancement that can circumvent this issue using the Laplacian operator is a viable alternative [30], given by:

(4) $$Y^{\prime }=\kappa \left(v\right)\cdot \left(h_{v}*Y+h_{h}*Y\right)+Y$$

where *Y* denotes the luminance channel of the input image; $h_{v}$ and $h_{h}$ represent vertical and horizontal Laplacian operators, respectively; * stands for the convolution operation; κ(v) refers to the adaptive scaling factor calculated using the image’s local variance *v*, and $Y^{\prime }$ is the enhanced luminance. Ngo et al. [30] proposed a scaling factor that uses three distinct gains for clear, slightly degraded, and heavily degraded areas in previous work. However, this type of weighting scheme is prone to visual artifacts due to the abrupt transition between gain values. In this paper, we, therefore, develop a weighting scheme with a linear form. The formula for calculating the proposed scaling factor is in Equation (5), where $\left(v_{1},v_{2}\right)$ and $\left(\kappa_{1},\kappa_{2}\right)$ are user-defined parameters utilized to specify the range of linear transformation.

(5) $$\kappa \left(v\right)=\{\begin{array}{cc} \kappa_{1} & v<v_{1}\\ \frac{v_{2}\kappa_{1}-v_{1}\kappa_{2}}{v_{2}-v_{1}}-\left(\frac{\kappa_{1}-\kappa_{2}}{v_{2}-v_{1}}\right)\cdot v & v_{1}\leq v\leq v_{2}.\\ \kappa_{2} & v>v_{2} \end{array}$$

**Figure 5:**
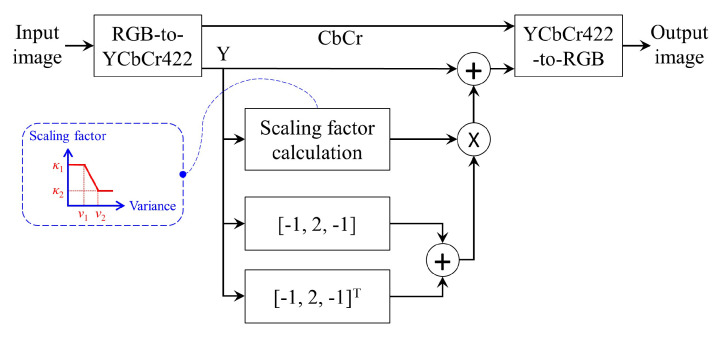
Block diagram of the detail enhancement module.

Figure 5 illustrates the block diagram of the detail enhancement module. The input image is converted to the YCbCr color space to enhance the luminance channel. The reason for this is that this channel contains much more high-frequency edge information than that of each R, G, and B channel. In addition, the YCbCr 4:2:2 format is exploited instead of the standard YCbCr 4:4:4 for reducing computing resources related to the algorithm’s data movement. Subsequently, the image data are converted back to the RGB color space for fetching to the gamma correction module.

### 3.2. Gamma Correction

**Figure 6:**
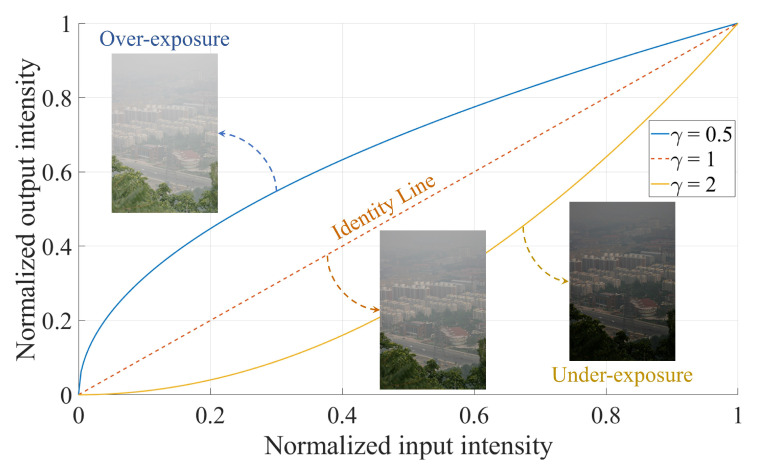
Artificial exposure by gamma correction.

To obtain images with different exposures, either the shutter speed or lens aperture must be adjusted to control the amount of light reaching the image sensors. However, these actions cannot be performed automatically as they are pertinent to physical devices. Thus, in this paper, we exploit a simple technique named gamma correction to artificially under-expose the captured image. This is a nonlinear image processing operation that is usually defined by the following power-law expression:

(6) $$I_{u}^{c}\left(x\right)={\left[I^{c}\left(x\right)\right]}^{\gamma }$$

where the super-script *c* denotes a color channel of the input image (i.e., c∈{R,G,B}), the subscript *u* represents under-exposure, and γ is a constant representing the exposure degree. Given the normalized image data of the range [0,1], over-exposure and under-exposure are represented by γ<1 and γ>1, respectively, as depicted in Figure 6. Specifically, γ=1 represents the ’identity line’, where the input intensity is left unchanged. Let *K* be the number of artificially under-exposed images generated by gamma correction. The empirical values for the corresponding set of $\gamma_{i},i\in \left[1,K\right]$ must satisfy $\gamma_{i}\geq 1$. Section 4.1 will delve deeply into the empirical settings of employed parameters.

### 3.3. Weight Calculation and Normalization

The selection of an appropriate weighting scheme in image fusion depends on the purpose of the designed algorithm. For example, Mertens et al. [37] utilized three image quality measures, including saturation, contrast, and well-exposedness. The reason for this is that their proposed algorithm fused a sequence of multi-exposure images into a high-quality image. Galdran [36] employed saturation and contrast in his fusion-based dehazing algorithm because these two features correlate with the haze distribution to a certain extent. In this paper, based on the comprehensive evaluation conducted by Ancuti et al. [38], the well-performed dark channel prior discovered by He et al. [7] lies a firm base for deriving a haze-aware weighting scheme.

Through extensive observations of clear images, He et al. [7] found that the clear visibility and colorfulness of captured scenes lead to the existence of dark pixels, whose intensity is close to zero on at least one color channel. This observation was then applied in a patch-based manner to define the dark channel, as follows:

(7) $$I^{DCP}\left(x\right)=min_{y\in \Omega (x)}\left\{min_{c\in \{R,G,B\}}\left[I^{c}\left(y\right)\right]\right\}$$

where *y* stands for pixel coordinates within the square window Ω(x) centered at *x*. From Equation (7), it is clear that haze-free patches possess extremely low DCP, while hazy patches exhibit large DCP due to the hazy effects. However, as the sky region has considerably high values in all its color channels, DCP does not hold. This problem is its widely recognized drawback and is left aside for now. It is necessary to inverse Equation (7) to develop a weighting scheme from DCP so that haze-free patches are assigned large weights and vice versa. Also, the previously mentioned shortcoming of DCP gives rise to the assignment of small weights to sky regions even though they are not obscured by haze; therein lies the cause of darkened sky after image fusion, as can be seen in Figure 4. To solve this problem, a post-processing step, which will be described in Section 3.5, is employed to judiciously enhance the luminance and color of the fused image. Formulas for the DCP-based weighting scheme as well as weight normalization are shown in Equations (8) and (9), respectively, where normalization is carried out to prevent an out-of-range problem.

(8) $$W^{DCP}\left(x\right)=1-I^{DCP}\left(x\right)$$

(9) $$W_{i}\left(x\right)=\frac{W_{i}^{DCP}\left(x\right)}{\sum_{i=1}^{K}W_{i}^{DCP}\left(x\right)}$$

Additionally, Galdran [36] stated that DCP was more suitable than a combination of saturation and contrast to guide the fusion process in haze removal. However, it was also assumed that DCP was not computationally friendly because it is usually post-processed by a large guided image filter. This is only applicable to cases of large Ω(x) (e.g., 15×15) whose block artifacts are noticeable. In this paper, the block artifacts are negligible for small Ω(x) (e.g., 3×3), so that the guided image filter can be excluded. Therefore, a 3×3 minimum filter and a simple multiplexing circuit suffice to compute the DCP. Conversely, in Galdran’s method [36], a 3×3 Laplacian filter and a complex square rooter are required for computing contrast and saturation. Thus, the proposed DCP-based weighting scheme is both computationally efficient and beneficial for dehazing.

### 3.4. Image Fusion

Because the multi-scale fusion based on the Laplacian pyramid is costly in terms of memory usage, in this paper, image fusion is conducted at a single scale as a simple weighted sum of under-exposed images and corresponding weight maps. In Equation (10), J is the dehazed image, $I_{u}^{i}$ is one of the under-exposed images $\left\{I_{u}^{1},I_{u}^{2},\ldots ,I_{u}^{K}\right\}$, and $W_{i}$ is one of the corresponding weight maps $\left\{W_{1},W_{2},\ldots ,W_{K}\right\}$.

(10) $$J\left(x\right)=\sum_{i=1}^{K}W_{i}\left(x\right)\cdot I_{u}^{i}\left(x\right)$$

**Figure 7:**
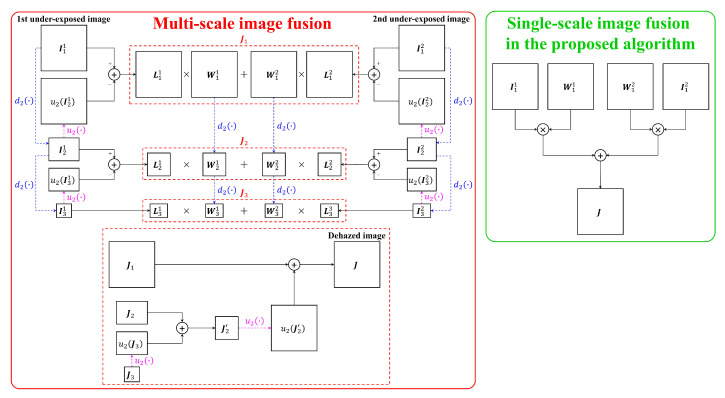
A visual illustration of multi-scale image fusion and single-scale image fusion employed in the proposed algorithm.

A detailed interpretation is then in order to support the use of single-scale image fusion. Assuming that there are two under-exposed images $\left\{I_{1}^{1},I_{1}^{2}\right\}$ derived from the single input image, two corresponding weight maps $\left\{W_{1}^{1},W_{1}^{2}\right\}$ are calculated based on the dark channel prior. In Figure 7, $u_{2}\left(\cdot \right)$ and $d_{2}\left(\cdot \right)$ denote the up-sampling and down-sampling operations by a factor of two, respectively. Moreover, the number of times to apply $u_{2}\left(\cdot \right)$ and $d_{2}\left(\cdot \right)$ is limited to two for simplicity. Accordingly, applying the Laplacian decomposition to $I_{1}^{1}$ and $I_{1}^{2}$ results in two sets $\left\{L_{1}^{1},L_{2}^{1},L_{3}^{1}\right\}$ and $\left\{L_{1}^{2},L_{2}^{2},L_{3}^{2}\right\}$. As shown in Figure 7, performing image fusion in the multi-scale manner is quite involved because the weighted sum operations are performed on individual scales to calculate the fused image through the final step including up-samplings and summations. Also, from the hardware designer’s point of view, several image buffers and line memories are required for performing up-sampling and down-sampling operations. However, the single-scale image fusion scheme in the proposed algorithm is simple as it solely comprises multiplications and summation by excluding the sampling operations that require large memories.

The computational flows illustrated in Figure 7 were programmed in the MATLAB environment, and a simple evaluation was conducted to verify the performance of the two fusion schemes. The two input images $\left\{I_{1}^{1},I_{1}^{2}\right\}$ were created by applying gamma correction with $\left\{\gamma_{1},\gamma_{2}\right\}=\left\{1,2\right\}$ to a single hazy image in the FRIDA2 [39], O-HAZE [40], and I-HAZE [41] datasets. The corresponding weight maps $\left\{W_{1}^{1},W_{1}^{2}\right\}$ were calculated using Equations (7)–(9). Table 1 summarized the evaluation results, wherein descriptions of three employed metrics are available in Section 4.3. Table 1 demonstrated that the difference in performance is negligible (i.e., less than 0.5% for all three datasets) even though multi-scale image fusion is far more complicated than single-scale image fusion. This observation, coupled with the interpretation above, supports the use of single-scale image fusion.

### 3.5. Dynamic Range Extension

The darkened sky after image fusion requires luminance enhancement; additionally, the whole image needs to be enhanced. Although the use of under-exposed images is beneficial to haze removal, it brings about the unwanted side effect of significantly darkening the entire image. Thus, the adaptive tone remapping (ATR) algorithm proposed by Cho et al. [42] is employed to post-process the fused image. ATR is expressed by the following Equations:

(11) $$EL\left(x\right)=L\left(x\right)+G_{L}\left(x\right)\cdot W_{L}\left(x\right)$$

(12) $$EC\left(x\right)=C\left(x\right)+G_{C}\left(x\right)\cdot W_{C}\left(x\right)+0.5$$

where *L* and EL denote the input luminance and the enhanced luminance, respectively; $G_{L}$ is the luminance gain, and $W_{L}$ represents the adaptive luminance weight. A similar interpretation holds for Equation (12) for color emphasis, wherein the constant 0.5 is an offset because the chrominance is subtracted in advance by 0.5 to be zero-centered. ATR exploits the input luminance’s cumulative distribution function to locate the adaptive limit point, which constitutes the nonlinear power function $G_{L}$. $W_{L}$ takes on the form of a linear function where $W_{L}$ is the dependent variable, and *L* is the independent variable. Since performing the color emphasis depends on the enhancement degree of the luminance, $G_{C}$’s formula is the multiplication of the ratio EL/L and the input color *C*. The last one, $W_{C}$, is a piece-wise linear function comprising three line segments. Interested readers are referred to Cho et al. [42] for a more detailed explanation.

## 4. Experiments

This section presents a comparative evaluation of the proposed algorithm and four benchmarking methods, including those proposed by He et al. [7], Zhu et al. [15], Kim et al. [15], and Galdran [36]. As mentioned in Section 1, although the recent method proposed by Ngo et al. [21] is quite efficient in image quality, its costly computations require considerable effort for future research. Therefore, it is better to exclude this algorithm from our list of benchmarking methods. However, we will demonstrate later in Section 4.3 that the proposed method is comparable to that of Ngo et al. [21] using their reported results.

The evaluation involves both a synthetic dataset and real datasets for thorough performance verification. FRIDA2 is a computer graphic-generated dataset designed for advanced driver-assistance systems, and it consists of 66 ground-truth images of road scenes. These images, coupled with their corresponding depth map, produce 264 hazy images covering four different haze types—homogeneous, heterogeneous, cloudy homogeneous, and cloudy heterogeneous. O-HAZE and I-HAZE are employed to assess the dehazing performance on real datasets. While O-HAZE comprises 45 pairs of outdoor hazy and haze-free images, I-HAZE comprises 30 pairs of indoor hazy and haze-free images. The hazy effects were simulated by a specialized vapor generator.

### 4.1. Experimental Setup

The proposed algorithm and four benchmarking methods were all programmed in MATLAB R2018b and tested on a computer with an Intel Core i5-7500 (3.4 GHz) CPU and 16 GB RAM. Default settings publicly provided by He et al. [7], Zhu et al. [18], Kim et al. [15], and Galdran [36] are those resulting in the best performance, as mentioned in their study. Thus, we used these parameter settings to configure the corresponding algorithms. Table 2 presents the empirically determined values of user-defined parameters employed in our work.

Generally, utilizing more under-exposed images increases the dehazing performance. However, because our ultimate goal is to provide a real-time IP of the proposed algorithm, the number of under-exposed images (i.e., *K*) is constrained by the limited hardware resources. As a result, K=4 is a feasible setting that maintains the hardware design’s simplicity. Concerning the detail enhancement step, the determined values of $\left\{v_{1},v_{2}\right\}$ must best divide a hazy image into three separate regions: (i) dense haze region whose local variance is less than $v_{1}$, (ii) moderate haze region whose local variance lies between $v_{1}$ and $v_{2}$, and (iii) haze-free region whose local variance is greater than $v_{2}$. For this reason, we have empirically chosen $\left\{v_{1},v_{2}\right\}=\left\{0.001,0.010\right\}$. After that, $\left\{\kappa_{1},\kappa_{2}\right\}$ were set to $\left\{2.500,1.000\right\}$ to enhance the detail information according to the piece-wise scaling factor in Equation (5). The reason for this is that when $\kappa_{1}$ was greater than 2.5, over-enhanced pixels appeared clipped whites, resulting in image quality degradation. Finally, four gamma values in the gamma correction step were determined based on two observations: (i) the set of under-exposed images should contain the hazy input, and (ii) the gamma value must be not too high to compensate for the limited hardware resources. Thus, $\left\{\gamma_{1},\gamma_{2},\gamma_{3},\gamma_{4}\right\}=\left\{1.000,1.900,1.950,2.000\right\}$ is a feasible setting.

### 4.2. Qualitative Evaluation

**Figure 8:**
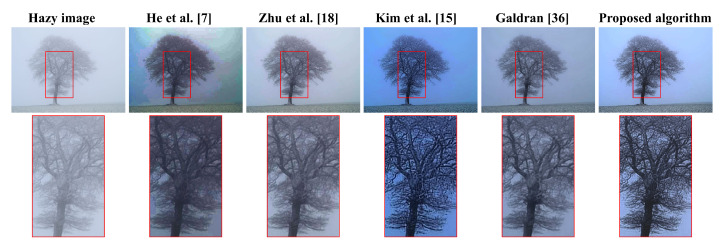
Qualitative comparison with other dehazing methods on a real hazy scene of a tree.

Figure 8 illustrates a real hazy scene of a tree obscured by moderate haze, to visually assess the five algorithms’ dehazing power. Additionally, these algorithms will be interchangeably referred to by the corresponding author list hereafter. Figure 8 demonstrated that algorithms proposed by He et al. [7], Zhu et al. [18], Kim et al. [15], and Galdran [36] had limitations. He et al. [7] suffers from visual artifacts in the background, Zhu et al. [18] exhibits too-weak dehazing power, Kim et al. [15] has a significant drawback of color distortion, and Galdran [36] appears to be a slightly under-exposed version of Zhu et al. [18] because the block-based contrast limited adaptive histogram equalization (CLAHE) employed therein does not bring about significant enhancement. In contrast, the proposed algorithm produces a satisfactory result due to the effective use of detail enhancement before image under-exposure and a DCP-based weighting scheme to guide the fusion process. Additionally, the well-known weakness of DCP in He et al. [7] and the darkening effect due to the use of under-exposed images are effectively resolved through adaptive tone remapping in post-processing.

**Figure 9:**
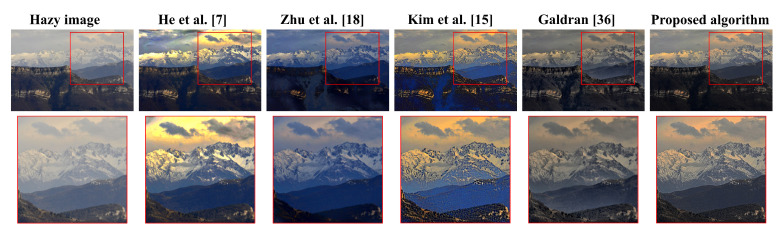
Qualitative comparison with other dehazing methods on a real hazy mountainous scene.

**Figure 10:**
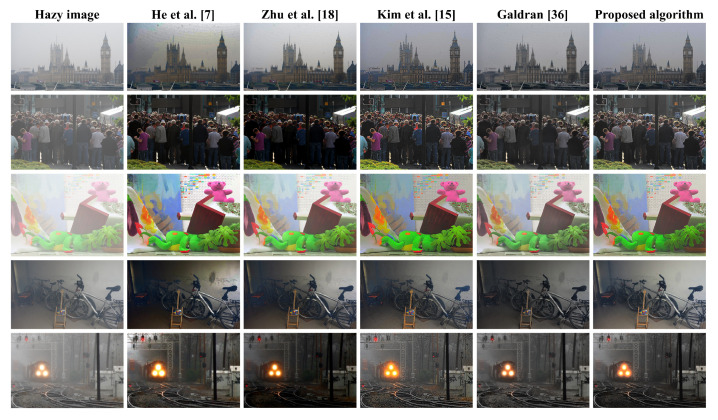
Qualitative comparison with other dehazing methods on various real hazy scenes.

Our method’s superior performance is confirmed in Figure 9, which depicts a mountain’s real hazy scene. Likewise, He et al. [7] suffers from visual artifacts including the yellowish sky and the bluish mountain, Zhu et al. [18] turns both the sky and the mountain bluish, Kim et al. [15] exhibits color distortion in the mountains, and Galdran [36] leaves a small portion of haze on the profile of the mountain. Only is ours capable of both removing the hazy effects and assuring high image quality. Figure 10 further demonstrates the proposed method’s dehazing performance with other benchmarking methods on various real hazy scenes. More evaluation results can be found online at: https://datngo.webstarts.com/blog/.

### 4.3. Quantitative Evaluation

This section utilized three evaluation metrics, including structural similarity (SSIM) [43], tone-mapped image quality index (TMQI) [44], and feature similarity extended to color images (FSIMc) [45], to access the five algorithms quantitatively. SSIM takes the luminance of both a dehazed image and a ground-truth reference as inputs and produces a value in [0,1] representing the degree of similarity in structural information. A higher SSIM implies a greater degree of similarity. Supposing that *X* and *Y* denote the dehazed and ground-truth reference images’ luminance channel, respectively, Equation (13) demonstrates the calculation of the SSIM measure of two images.

(13) $$SSIM\left(X,Y\right)=\frac{\left(2\mu_{x}\mu_{y}+C_{1}\right)\left(2\sigma_{xy}+C_{2}\right)}{\left(\mu_{x}^{2}+\mu_{y}^{2}+C_{1}\right)\left(\sigma_{x}^{2}+\sigma_{y}^{2}+C_{2}\right)}$$

where $\left(\mu_{x},\mu_{y}\right)$ and $\left(\sigma_{x},\sigma_{y}\right)$ represent the local average and standard deviation of *X* and *Y*, respectively; $\sigma_{xy}$ denotes the correlation coefficient between the mean-subtracted $\left(X-\mu_{x}\right)$ and $\left(Y-\mu_{y}\right)$, and $C_{1}$ and $C_{2}$ are stabilizing constants.

The second metric, TMQI, works on the luminance of images and assesses the multi-scale fidelity measure based on the structural fidelity (*S*) and the naturalness (*N*), as presented in Equation (14). Wherein 0≤a≤1 is a constant to adjust the relative importance of the two terms, and φ and ϕ are exponents to control their corresponding sensitivities. As with the SSIM mentioned above, *X* and *Y* denote the dehazed and ground-truth reference images.

(14) $$TMQI\left(X,Y\right)=aS^{\varphi }+\left(1-a\right)N^{\phi }$$

The structural fidelity is calculated based on the modified SSIM ($S_{local}$), as shown in Equations (15) and (16). In these two equations, $x_{i}$ and $y_{i}$ are the *i*-th local patches in the two images *X* and *Y*, respectively; $P_{q}$ is the number of local patches in the *q*-th scale; $\psi_{q}$ is the weight corresponding to the *q*-th scale, and *Q* is the total number of scales. $\sigma_{x}$ and $\sigma_{y}$ are passed through the nonlinear sigmoid function to produce the mapped $\sigma_{x}^{{}^{\prime }}$ and $\sigma_{y}^{{}^{\prime }}$. The reason for this is to consider the visual sensitivity of contrast in the literature of visual psychophysics.

(15) $$S=\prod_{q=1}^{Q}{\left[\frac{1}{P_{q}}\sum_{i=1}^{P_{q}}S_{local}\left(x_{i},y_{i}\right)\right]}^{\psi_{q}}$$

(16) $$S_{local}\left(x,y\right)=\frac{\left(2\sigma_{x}^{{}^{\prime }}\sigma_{y}^{{}^{\prime }}+C_{1}\right)\left(\sigma_{xy}+C_{2}\right)}{\left({\sigma_{x}^{{}^{\prime }}}^{2}+{\sigma_{y}^{{}^{\prime }}}^{2}+C_{1}\right)\left(\sigma_{x}\sigma_{y}+C_{2}\right)}$$

To calculate the naturalness, Yeganeh et al. [44] first fitted the means and standard deviations of 3000 gray-scale images to the Gaussian and Beta distributions. Then, they defined the naturalness measure as follows:

(17) $$N=\frac{P_{m}P_{d}}{Z}$$

where $P_{m}$ and $P_{d}$ denote the Gaussian and Beta probability density functions, respectively, and $Z=max\left(P_{m}P_{d}\right)$ denotes the normalization factor to constrain *N* between 0 and 1. Since both the structural fidelity and the naturalness are upper-bounded by 1, TMQI is also upper-bounded by 1, wherein a higher score is favorable to haze removal.

The third metric, FSIMc, can be considered an improvement upon SSIM since it extends its calculation to the chrominance. Equation (18) demonstrates the calculation of FSIMc, wherein *X* and *Y* are now two color images, that is, a dehazed image and a ground-truth reference image, $S_{L}$ is the combined similarity measure of the gradient magnitude and the phase congruency similarities between *X* and *Y*, $S_{C}$ is the chrominance similarity measure, $PC_{m}$ is the weighting coefficient, Γ is a positive constant for adjusting the importance of the chrominance component, and Ω is the whole image domain. FSIMc also takes on values between 0 and 1, wherein a higher score implies a better dehazing performance.

(18) $$FSIMc\left(X,Y\right)=\frac{\sum_{i\in \Omega }S_{L}\left(i\right)S_{C}{\left(i\right)}^{\Gamma }PC_{m}\left(i\right)}{\sum_{i\in \Omega }PC_{m}\left(i\right)}$$

Table 3 and Table 4 show quantitative evaluation results on the FRIDA2, O-HAZE, and I-HAZE datasets, respectively, where the best results appear in boldface. Regarding the synthetic FRIDA2 dataset, Table 3 demonstrated that the proposed algorithm exhibits the best dehazing power in terms of SSIM and FSIMc. The employed detail enhancement and ATR post-processing primarily contribute to the high SSIM and FSIMc scores. More specifically, the former accentuates the objects’ profile, and the latter performs both luminance enhancement and color emphasis. The fact that He et al. [7] shows the lowest performance is owing to the contents of the FRIDA2 dataset, which comprises road scenes covered by a broad sky. Zhu et al. [18] is better than He et al. [7], albeit unsatisfactory owing to the drawbacks of over-dehazing, background noise, and color distortion, as mentioned in Reference [19]. Likewise, Kim et al. [15] is slightly better than He et al. [7], mainly due to the modified hybrid median filter for estimating the atmospheric veil. However, as pointed out in Reference [19], Kim et al. [15] is prone to noticeable background noise and color distortion. Galdran [36] shares top performance with the proposed method, and its TMQI is the highest, which is primarily due to the block-based CLAHE’s preference for the multi-scale base for TMQI. However, for the O-HAZE dataset, this observation is reversed. Galdran [36] is the best method under SSIM and FSIMc, while ours exhibits the best performance under TMQI. Dataset dependence can be responsible for this observation. Since O-HAZE consists of outdoor images with homogeneous lighting conditions, the block-based CLAHE can bring about a significant enhancement in image contrast without any noticeable artifacts, subsequently resulting in the top performance of Galdran [36]. Nevertheless, as the I-HAZE dataset comprises indoor images with heterogeneous lighting conditions, the proposed method is interestingly superior to the four benchmarking ones in all three employed metrics.

To conclude this section, a brief comparison of the proposed method with Ngo et al. [21] is in order. According to their reported results, the pairs of {SSIM, TMQI} scores were {0.7297,0.7993}, {0.7630,0.8244}, and {0.7727,8522} on FRIDA2, O-HAZE, and I-HAZE datasets, respectively. It is evident from Table 3 and Table 4 that the proposed method always possesses slightly higher scores than that of Ngo et al. [21], albeit with a significantly lower computational cost.

## 5. Real-Time Processing

In this section, a hardware architecture for the proposed algorithm is presented for facilitating real-time processing. The synthesis results then prove that the proposed hardware design is compact and capable of handling high-quality videos in real-time.

### 5.1. Hardware Implementation

The hardware architecture for implementing the proposed algorithm in a Field Programmable Gate Array (FPGA) chip [46] is described in the same order as presented in Section 3. The hazy input is first fetched to the detail enhancement module, in which color conversion (i.e., RGB to YCbCr 4:2:2 and vice versa) is realized by using basic arithmetic operations. However, it should be noted that multiplication comprising too large multiplicands and multipliers (e.g., bit sizes greater than 16) is accomplished by means of split multipliers, which exploit associative and distributive properties to achieve throughput improvement of hardware real-time maximum frequency. The division in Equation (5) is implemented by serial dividers as user-defined parameters do not change from pixel to pixel, like the real-time input image data.

**Figure 11:**
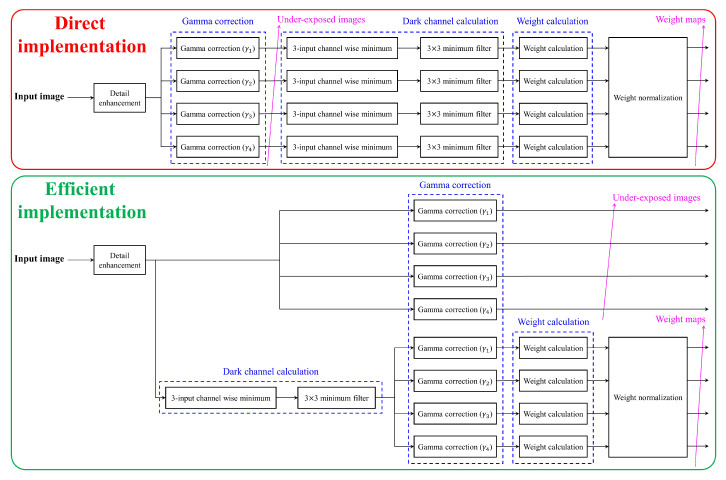
The proposed efficient implementation for calculating weight maps by exploiting the monotonicity of gamma correction.

**Figure 12:**
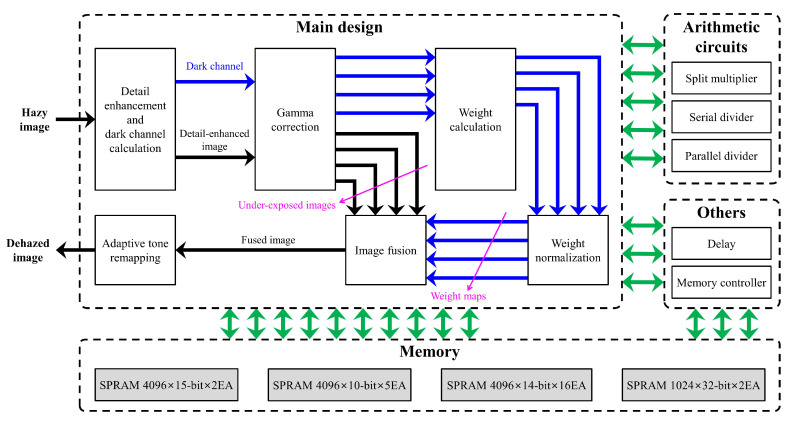
Hardware architecture of the proposed algorithm.

The detail-enhanced images are subjected to gamma correction for generating under-exposed images, where Equation (6) is realized by an efficient means of look-up tables (LUTs). The minimum filter in weight calculation and the vertical Laplacian operator in detail enhancement involve convolution, so they are implemented using line memories and their corresponding memory controllers. In this study, the proposed hardware is designed to handle the maximum video resolution of 4K. Thus, each line memory is designed for 4096 pixels, giving rise to the need for an efficient design to calculate weight maps. Sequentially following Equations (6)–(9) result in a direct implementation illustrated in the top half of Figure 11. Because four under-exposed images are generated, four sets of a 3-input channel-wise minimum followed by a 3×3 minimum filter are required to find the dark channels in Equation (7). Accordingly, eight line memories (two for each minimum filter times four filters) are utilized solely for calculating the dark channels. In an attempt to reduce the number of line memories, the monotonicity of Equation (6) is worthy of attention. The gamma correction is a monotonically decreasing operation for γ≥1, so that the location of minimum pixels in all three color channels and local patches are preserved. Hence, the efficient implementation in the bottom half of Figure 11 exploits the monotonicity to reduce the number of line memories to two at the cost of doubling the number of LUTs. However, utilizing more LUTs is not problematic because LUT is considerably simpler than line memory. Consequently, by calculating weight maps via the efficient implementation in the proposed algorithm, the number of requisite line memories has been reduced from eight to two, that is 75% more efficient than the direct implementation in terms of memory usage. In Figure 11, it is worth noticing that delay modules ensuring the correct pipelined operations have been omitted for ease of illustration. Also, the set of a 3-input channel-wise minimum followed by a 3×3 minimum filter is included in the detail enhancement module, and two sets of gamma correction LUTs are also represented as a single gamma correction module in Figure 12, which shows the whole hardware architecture.

Furthermore, because the real-time input data are required to be processed continuously, the division in Equation (9) for weight normalization is achieved by means of parallel dividers. Image fusion on a single scale is simply accomplished by using multipliers and adders. Finally, the IP for the ATR designed by Cho et al. [42] is exploited to post-process the fused image.

### 5.2. Synthesis and Comparison

The hardware architecture proposed in Figure 12 was designed using the Verilog hardware description language (IEEE Standard 1364-2005) [47] and synthesized using a Xilinx Design Analyzer. The synthesis results are summarized in Table 5. Our design utilized 30,676 registers, 36,357 LUTs, and 48 block RAMs. This occupies 7.02%, 16.63%, and 8.81% of available resources on the target FPGA chip, respectively. The maximum attainable processing rate is 242.718 MHz or Mpixels/s. Using this information, the maximum processing speed (MPS) in frames per second (fps) can be derived as follows:

(19) $$MPS=\frac{f_{max}}{\left(W+HB)\cdot (H+VB\right)}$$

where $f_{max}$ denotes the maximum operating frequency, *W* and *H* represent the width and height of the input image, respectively, and HB and VB represent the corresponding horizontal and vertical periods. The proposed hardware is designed so that it functions properly with the minimum HB and VB of one pixel and one line, respectively. Table 6 presents the maximum processing speeds for different video resolutions, demonstrating that our hardware implementation is capable of handling DCI 4K video at 27 fps. More specifically, 8,853,617 (=4097×2161) clock cycles are required for processing one video frame of size 4096×2160. Therefore, substituting this value to Equation (19) results in the MPS of 27 (≈$242.718\times 10^{6}/$8,853,617).

Table 7 shows the synthesis results of the proposed design and the other two methods side-by-side to illustrate the compactness and efficiency of our dehazing hardware. Park et al. [48] developed a fast execution scheme of the algorithm proposed by He et al. [7]. Their design comprises 53,400 registers, 64,000 LUTs, 32 digital signal processing (DSP) slices and 3.2 Mbits. Notwithstanding the maximum processing rate of 88.700 Mpixels/s, the hardware was designed to operate at fixed frame sizes of 320×240, 640×480 and 800×600. Accordingly, the maximum video resolution that the design of Park et al. [48] can handle is solely super video graphics array (SVGA). Ngo et al. [17] developed a 4K-capable IP of the algorithm proposed by Kim et al. [15], and it consists of 70,864 registers, 56,664 LUTs and 1.5 Mbits. The highest possible attainable processing speed is 236.290 MHz, which is responsible for its 4K capability. Compared to these two designs, our haze removal hardware is quite compact and fast. More specifically, when compared to the design of Park et al. [48], the utilization of registers, LUTs, and memory is reduced by 42.6%, 43.2% and 59.4%, respectively; and the processing speed is increased by approximately thrice. Compared to the design of Ngo et al. [17], the reduction rates in the utilized registers, LUTs, and memory are 56.7%, 35.8% and 13.3%, respectively, and the processing speed is slightly improved. Thus, coupled with the algorithm performance evaluation in Section 4, it can be concluded that our proposed algorithm is of paramount importance because of its superior performance and an efficient hardware prototype that is capable of handling high-quality video streams in real-time.

## 6. Conclusions

A computationally efficient haze removal algorithm and its corresponding hardware implementation were presented in this paper. It was discovered that dehazing methods based on the Koschmieder model are computationally expensive, mainly due to the inevitable estimation process of unknown variables, that is, transmission map and atmospheric light. Therefore, we first exploited Koschmieder’s law to deduce the use of under-exposed images for haze removal. Then, simple image processing techniques such as detail enhancement, gamma correction, single-scale image fusion, and adaptive tone remapping were employed to carry our deduction into effect. The use of detail enhancement before artificial under-exposure by gamma correction effectively mimicked the physical exposure adjustment, while the DCP-based weighting scheme accurately guided the fusion process to blend image areas with clear visibility into the fused image. A novel adaptive tone remapping algorithm enhanced the darkened result obtained after fusing under-exposed images. Moreover, a compact hardware design capable of processing DCI 4K video standard was provided to facilitate the integration of the proposed method into existing real-time systems.

References yes

## Figures and Tables

**Table 1:** Average structural similarity (SSIM), tone-mapped image quality index (TMQI), and feature similarity extended to color images (FSIMc) scores on the FRIDA2, O-HAZE, and I-HAZE datasets for evaluating multi-scale and single-scale image fusions.

Dataset	Fusion Scheme	SSIM	TMQI	FSIMc
	Multi-scale	0.7431	0.6800	0.8002
FRIDA2	Single-scale	0.7428	0.6795	0.8000
	Difference (%)	0.0404	0.0735	0.0250
	Multi-scale	0.6799	0.8184	0.7551
O-HAZE	Single-scale	0.6768	0.8172	0.7529
	Difference (%)	0.4559	0.1466	0.2914
	Multi-scale	0.7170	0.7397	0.8104
I-HAZE	Single-scale	0.7159	0.7389	0.8096
	Difference (%)	0.1534	0.1082	0.0987

**Table 2:** Empirical values of user-defined parameters in the proposed algorithm.

Parameter	Description	Value
*K*	The number of under-exposed images	4
$\left\{v_{1},v_{2},\kappa_{1},\kappa_{2}\right\}$	Being used to control detail enhancement step	{0.001,0.010,2.500,1.000}
$\left\{\gamma_{1},\gamma_{2},\gamma_{3},\gamma_{4}\right\}$	Gamma values in gamma correction step	{1.000,1.900,1.950,2.000}

**Table 3:** Average SSIM, TMQI, and FSIMc scores on FRIDA2 dataset. The boldface numbers indicate the best performance.

Method	Haze Type	SSIM	TMQI	FSIMc
He et al. [7]	Homogeneous	0.6653	0.7639	0.8168
	Heterogeneous	0.5374	0.6894	0.7251
	Cloudy Homogeneous	0.5349	0.6849	0.7222
	Cloudy Heterogeneous	0.6500	0.7781	0.8343
	Overall Average	0.5969	0.7291	0.7746
Zhu et al. [18]	Homogeneous	0.5651	0.7533	0.7947
	Heterogeneous	0.5519	0.7254	0.7845
	Cloudy Homogeneous	0.5310	0.7080	0.7764
	Cloudy Heterogeneous	0.5412	0.7674	0.8117
	Overall Average	0.5473	0.7385	0.7918
Kim et al. [15]	Homogeneous	0.5949	0.7320	0.8048
	Heterogeneous	0.6245	0.7037	0.7805
	Cloudy Homogeneous	0.6124	0.7015	0.7751
	Cloudy Heterogeneous	0.6078	0.7343	0.8135
	Overall Average	0.6099	0.7179	0.7935
Galdran [36]	Homogeneous	0.7200	0.7397	0.7958
	Heterogeneous	0.7213	0.7436	0.7909
	Cloudy Homogeneous	0.6921	0.7250	0.7800
	Cloudy Heterogeneous	0.7595	0.7588	0.8183
	Overall Average	0.7232	**0.7418**	0.7963
Proposed Algorithm	Homogeneous	0.7545	0.7295	0.8125
	Heterogeneous	0.7345	0.7204	0.7991
	Cloudy Homogeneous	0.7423	0.7235	0.7963
	Cloudy Heterogeneous	0.7278	0.7172	0.7902
	Overall Average	**0.7398**	0.7227	**0.7995**

**Table 4:** Average SSIM, TMQI, and FSIMc scores on O-HAZE and I-HAZE datasets. The boldface numbers indicate the best performance.

Dataset	Method	SSIM	TMQI	FSIMc
	He et al. [7]	0.7709	0.8403	0.8423
	Zhu et al. [18]	0.6647	0.8118	0.7738
O-HAZE	Kim et al. [15]	0.4702	0.6509	0.6869
	Galdran [36]	**0.7877**	0.8401	**0.8468**
	Proposed Algorithm	0.7753	**0.8991**	0.8350
	He et al. [7]	0.6580	0.7319	0.8208
	Zhu et al. [18]	0.6864	0.7512	0.8252
I-HAZE	Kim et al. [15]	0.6424	0.7026	0.7879
	Galdran [36]	0.7547	0.7613	0.8558
	Proposed Algorithm	**0.7779**	**0.8077**	**0.8583**

**Table 5:** Hardware synthesis result of the proposed hardware design.

Xilinx Design Analyzer ${}^{1}$			
Device	xc7z045-2ffg900		
Slice Logic Utilization	Available	Used	Utilization
Slice Registers (#)	437,200	30,676	7.02%
Slice LUTs (#)	218,600	36,357	16.63%
Used as Memory (#)	70,400	529	0.75%
RAM36E1/FIFO36E1s	545	48	8.81%
Minimum Period	4.120 ns		
Maximum Frequency	242.718 MHz		

${}^{1}$ The EDA Tool was supported by the IC Design Education Center.

**Table 6:** Maximum processing rate for various video resolutions.

Video Resolution		Frame Size	Required Clock Cycles (#)	Processing Speed (MPS)
Full HD (FHD)		1920×1080	2,076,601	116
Quad HD (QHD)		2560×1440	3,690,401	65
	UW4K	3840×1600	6,149,441	39
4K	UHD TV	3840×2160	8,300,401	29
	DCI 4K	4096×2160	8,853,617	27

**Table 7:** Comparison with other hardware designs.

Hardware Utilization	Park et al. [48]	Ngo et al. [17]	Proposed Design
Registers (#)	53,400	70,864	30,676
LUTs (#)	64,000	56,664	36,357
DSPs (#)	42	0	0
Memory (Mbits)	3.2	1.5	1.3
Maximum Frequency (MHz)	88.700	236.290	242.718
Maximum Video Resolution	SVGA	DCI 4K	DCI 4K
